# Expression of concern: The turnaway study: A case of self-correction in science upended by political motivation and unvetted findings

**DOI:** 10.3389/fpsyg.2022.1049195

**Published:** 2022-10-05

**Authors:** Frontiers Editorial Office

**Keywords:** abortion, mental health, turnaway study, bias, politicized science

With this expression of concern, Frontiers acknowledges complaints received regarding this article. This statement will remain while an investigation is carried out in full accordance with our procedures. It will be updated, and any necessary further action will be taken, at the conclusion of the investigation.

